# Left ventricular systolic function in dogs with pulmonic stenosis

**DOI:** 10.14202/vetworld.2020.2436-2442

**Published:** 2020-11-14

**Authors:** Ploypanut Trikhun, Sirilak Disatian Surachetpong, Saikaew Sutayatram, Chollada Buranakarl

**Affiliations:** 1 Department of Physiology, Faculty of Veterinary Science, Chulalongkorn University, Henri Dunant Road, Pathumwan, Bangkok, 10330, Thailand; 2 Department of Medicine, Faculty of Veterinary Science, Chulalongkorn University, Henri Dunant Road, Pathumwan, Bangkok 10330, Thailand

**Keywords:** dogs, pulmonic stenosis, ventricular function

## Abstract

**Background and Aim:**

Pulmonic stenosis (PS) is the most common congenital heart disease in dogs. This condition causes right ventricle (RV) overload and disrupts overall systolic function. The aim of this study was to examine the alterations of cardiac electrical activity and mechanical function in dogs with PS compared to normal healthy dogs.

**Materials and Methods:**

The ventricular systolic function of dogs with PS was studied. Dogs were divided into two groups, PS (n=13) and control (CONT) (n=12). Measurements of blood pressure, electrocardiography (ECG), and echocardiography were performed.

**Results:**

PS dogs had exercise intolerance, with six experiencing syncope. ECG of PS dogs showed higher amplitudes of P, S, and T waves (p<0.01), and a lower R:S ratio (p<0.001) with longer QRS duration (p<0.001) compared to CONT dogs. Echocardiography demonstrated that the pulmonic flow velocity and pressure gradient (PG) between the RV and the pulmonary artery of PS dogs were significantly higher than CONT dogs (p<0.001). The RV free wall thickness to the left ventricular posterior wall thickness ratio and the right atrium to the left atrium diameter ratio was higher (p<0.001), while interventricular septum (IVS) was thicker (p<0.01) in PS dogs compared with CONT dogs (p<0.001). The systolic function in PS dogs showed higher pulmonic valve velocity time integral (PVVTI) value (p<0.001) and longer pulmonic valve ejection time (ET) (p<0.05) than CONT dogs. However, aortic valve VTI (AVVTI) value and aortic valve ET were not significantly different between the groups, although fractional shortening in PS dogs was higher. In PS dogs, PG showed a significant positive correlation with PVVTI: AVVTI ratio (p<0.05).

**Conclusion:**

PS had prolonged pulmonic valve opening. The thickening of the RV wall and IVS can cause a detrimental reduction of the left ventricular preload in PS dogs.

## Introduction

Pulmonic stenosis (PS) is a congenital narrowing of the pulmonic valve located between the right ventricle (RV) and the pulmonary artery (PA), which is the most common congenital heart disease in many breeds of dogs [1,2]. Balloon valvuloplasty is the modern day treatment of choice for the treatment of PS [3]. PS is classified depending on location, anatomy of valves, and severity of stenosis. The location of stenosis can be subvalvular, valvular, or supravalvular. By considering the anatomy of valve, PS can be further divided into types A and B, depending on the thickening and hypoplasia of the valve [4]. Moreover, the severity of PS is graded based on the pressure gradient (PG) across the RV and the RV outflow tract, as measured by echocardiography [4].

The most common clinical signs of PS in dog, depending on the severity, are exercise intolerance, syncope, and sudden death. A systolic murmur is also noted on the left heart base during physical examination. Ascites or pleural effusion is also possible outcomes for right-sided heart failure. The cardiac electrical abnormalities and cardiac structural alterations related to RV hypertrophy were demonstrated in dogs with PS [5,6]. Nevertheless, information on systolic function and the relationship between right and left sides of the heart has been limited.

The aim of this study was to investigate the differences in the electrical activity and systolic functions, including their hemodynamic significances, in dogs with PS compared to dogs without PS.

## Materials and Methods

### Ethical approval and informed consent

The protocol of this study was conducted in accordance with the standard animal use guidelines and the Institutional Animal Care and Use Committees (IACUC) (protocol No 1831072). The consent forms were signed by all owners.

### Animals

The client own dogs in the present study were obtained from the Small Animal Teaching Hospital, Faculty of Veterinary Science, Chulalongkorn University. This study consists of both retrospective and prospective studies from 2016 to 2019. The dogs were divided into two groups, PS group and control (CONT) group. The dogs were classified by breed and age, and those of CONT were matched to those of the PS group. The dogs in CONT were client-owned healthy dogs. This was established by a complete physical examination, blood profile analysis, chest radiography, electrocardiography (ECG) and echocardiography. All CONT dogs had negative results for heartworm antigen and negative antibodies for Ehrlichia, Anaplasma, and Borrelia burgdorferi sensu lato (Lyme disease) using the Idexx SNAP 4Dx^®^ test kit (IDEXX, Westbrook, ME, USA). The blood parasite was also negative from the light microscopic examination. In the PS group, PS was diagnosed by the presence of stenosis of the pulmonic valve regardless of location (valvular, subvalvular, or supravalvular), stenotic type (A or B), severity of disease (mild, moderate, or severe), or other coexisting congenital heart abnormities. The stenosis of pulmonic valves can also be present at one or multiple locations. The PS dogs with other systemic diseases and heartworm disease were excluded from the study.

### Experimental protocol

After history taking from the owners, both CONT (n=12) and PS dogs (n=13) were subjected to complete physical examination. Each dog was kept in a quiet room with minimal restraints while indirect blood pressure, ECG, and echocardiography were performed in that order.

#### Blood pressure measurement

Indirect blood pressure was measured when dogs were at rest using the oscillometric method (SunTech® Vet20, SunTech Medical, USA). The dogs were gently restrained in the right lateral recumbent position. The appropriate cuff size, using approximately 40% of the limb circumference, was applied on the left forelimb. Systolic blood pressure (SBP) was recorded 3 times, and the average blood pressure was recorded.

#### Electrocardiographic recording

The surface ECG recording was performed according to the standard veterinary procedure using an ECG machine (CariMax FX-7102, FUKUDA DENSHI, Japan). Dogs were restrained in the right lateral recumbent position without sedation and attached with four ECG electrode clips at all four legs for the limb leads including leads I, II, III, aVR, aVL, and aVF. Alcohol or ECG gel was applied at the electrode sites for electrical conduction. ECG signals were recorded for at least 30 s. The QTc was calculated from lead II according to Van de Water’s formula: QTcV=QT−0.087 (RR interval−1000) [7].

#### Conventional echocardiography measurement

The echocardiography procedure followed the standard procedure in veterinary practice. The dogs were examined by the same cardiologist to avoid inter-observer variation. The dogs were restrained on a table in the right lateral recumbent position without sedation. ECG electrode clips were applied on three limbs for lead II recording. The functional and hemodynamic parameters of the left cardiac function using two-dimensions (2D), M-mode, and Doppler techniques including pulse-wave Doppler and continuous-wave (CW) Dopplers were performed on an echocardiographic machine (EKO-7, Samsung Medison Co., Ltd., Gangnam-gu, Seoul, South Korea) with 2-4 and 4-12 multi-frequency MHz phased array transducers.

The 2D echocardiography was performed for the assessment of pulmonic valve abnormalities and PS type classification. The types of PS are classified as previously mentioned while the severity was categorized according to PG across pulmonic valves.

The M-mode echocardiography was performed for the evaluation of myocardial thickness and cardiac chamber diameters during systole and diastole on the right parasternal long-axis (PLAX) view. The parameters were interventricular septum (IVS) thickness during diastole (IVSd) and systole (IVSs), left ventricular internal diameter(LVID) during diastole (LVIDd) and systole (LVIDs), and left ventricular posterior (LVPW) thickness during diastole (LVPWd) and systole (LVPWs). Fractional shortening (FS) was retrieved from an echocardiographic software program according to the machine (EKO-7, Samsung Medison Co., Ltd., Gangnam-gu, Seoul, South Korea). The measurement of the diameters of the left atrium (LA) and aortic root (AO) and LA:AO was also performed during diastole in the right parasternal short axis (PSAX) view. RV thickening and right atrium (RA) enlargement were measured from M-mode echocardiography in the right PLAX view.

Color flow Doppler echocardiography was performed to identify abnormal flow associated with turbulence within the PA. The pulmonic flow velocity (PV) and PG were measured on the right PSAX view at the level of pulmonic valve. The PG across the stenotic part was calculated by the modified Bernoulli equation for PS severity grading system. In addition, CW Doppler was used to measure velocity time integral (VTI) and systolic time interval (STI) at valvular areas of the aortic or the pulmonic valves in the right PSAX view. The VTI can be calculated offline using a VTI program by manually lining the VTI cursors around the outlining of the blood flow across the valve. The STI values, including the pre-ejection period (PEP) and ejection time (ET), were generated by manually lining the starting time of PEP and the start and end of normal ET.

### Statistical analysis

Data are presented as mean±the standard error of the mean. Parameters obtained in the CONT dogs were compared with the PS dogs using the unpaired t-test or the Mann–Whitney U-test. The relationships between parameters were performed using the Pearson correlation. p<0.05 is considered as statistical significance.

## Results

### General characteristics and clinical presentation of dogs

The dogs in this study comprised two groups, CONT and PS groups. Twelve healthy dogs of seven intact males and five intact females composed the CONT dogs, while 13 dogs with PS – nine intact males and four intact females – composed the PS dogs. The average age and body weight of CONT and PS dogs were not different and shown in Table-1. Dogs ranged from 6 months old to 5 years old and 3.6 months old to 5 years old, while the body weights ranged from 4.1-26.0 kg to 4.4-24.0 kg in CONT and PS groups, respectively. The CONT group consisted of six French Bulldogs, two English Bulldogs, one Pomeranian, one Beagle, one Poodle, and one Shih Tzu while the PS group consisted of the same matched breeds except two Pomeranians. Other cardiac defects, including atrial septal defects (ASD) and coronary aberrance, were found in the PS group. Two French Bulldogs and one Pomeranian had additional ASD, one Bulldog had coronary aberrance, while one Bulldog had both.

**Table-1:** General characteristics in control and pulmonic stenosis dogs.

Parameters	CONT group	PS group
Age (year)	1.56±0.36	1.76±0.38
Body weight (kg)	11.20±1.87	9.38±1.47
Sex (M/F)	(7/5)	(9/4)
Breed	6 French Bulldogs	6 French Bulldogs
	2 English Bulldogs	2 English Bulldogs
	1 Pomeranian	2 Pomeranians
	1 Beagle	1 Beagle
	1 Poodle	1 Poodle
	1 Shih-tzu	1 Shih-tzu
Other congenital defects		3/13 PS with ASD
		1/13 PS with coronary aberrance
		1/13 PS with ASD and coronary aberrance

Data presented as mean±SEM. CONT=Control, PS=Pulmonic stenosis, M=Intact male, F=Intact female, ASD=Atrial septal defect

All PS dogs presented with exercise intolerance, while half of them had syncope. Polycythemia was found in two out of three dogs that had ASD. About four dogs (30%) in PS showed signs of the right side congestive heart failure such as ascites and jugular distension. Within these four dogs, three of them received pimobendan approximately 1 week before study, in addition to other drugs including furosemide, sildenafil, and combined furosemide and ramipril, respectively.

### Physical examination

Complete physical examination results revealed systolic murmur Grade 3-4 at the left heart base in 6 PS dogs. The average HR from auscultation was 120±7 beats per min (BPM) and 136±7 BPM, and the average RR was 47±4 and 49±4 breaths per min in CONT and PS groups, respectively, and were not significantly different between groups. The average SBP was 148±5 mmHg in CONT dogs and 150±7 mmHg in PS dogs, which were not significantly different between groups.

### ECG

The ECG results are shown in Table-2. Respiratory sinus arrhythmia (RSA) was found in all CONT dogs. Six dogs in PS group had sinus tachycardia (ST), while RSA and normal sinus rhythm were found in five and two dogs, respectively.

**Table-2:** The electrocardiogram findings and waveform amplitudes and durations in control and pulmonic stenosis dogs.

Parameters	CONT group	PS group
P wave amplitude (mV)	0.12±0.01	0.31±0.05**
R wave amplitude (mV)	1.01±0.11	0.56±0.08**
S wave amplitude (mV)	0.06±0.01	0.80±0.20**
R:S wave ratio	22.9±3.4	5.0±2.3***
T wave amplitude (mV)	0.16±0.03	0.36±0.08**
P duration (ms)	47.4±2.5	48.1±2.7
PR interval (ms)	83.5±4.2	76.2±4.1
QRS duration (ms)	51.1±2.3	73.8±4.4***
QT interval (ms)	220.4±8.1	266.1±35.5
QTc (ms)	297.0±8.4	352.1±35.5
Heart rate (BPM)	117±6	139±11

Data are presented as mean±SEM using t-test.

** p<0.01 and

*** p<0.001. CONT=Control, PS=Pulmonic stenosis, QTc=Corrected QT interval, mV=Millivolt, ms=Millisecond and BPM=Beats per minute

None of the ECG waveforms in CONT dogs showed negative deep S wave, while this was present in ten out of 13 PS dogs which suggested the presence of the right axis deviation in PS dogs. The amplitudes of the P, S, and T waves in the PS group were higher while the R wave was lower (p<0.01) and resulting in a lower R:S ratio (p<0.001). The QRS duration was longer in the PS group compared with the CONT group (p<0.001). The P duration, PR interval, QT interval, and QTc were similar between groups. The heart rate in the PS group was slightly higher but not significantly different when compared with the CONT group.

### Echocardiographic and Doppler study

The pulmonic valve abnormalities were classified depending on 2D echocardiographic examination results. Valvular stenosis was the most common abnormality in PS dogs in this study (76.9% or 10/13), while combined valvular with supravalvular stenosis was found in only 23.1% (3/13). Type A and type B PS were observed in 7 (53.8%) and 6 PS dogs (46.2%), respectively. The severity of PS consisted of ten severe, two moderate, and one mild PS, classified by PV and PG across RV and PA from echocardiographic measurement.

The results of normalized echocardiographic parameters are shown in Table-3. PS group had significantly increased IVSdN and IVSsN thickness (p<0.01) with decreased LVIDdN and LVIDsN diameters (p<0.001), as compared with CONT group when measured from M-mode of echocardiography. CW Doppler study indicated that PS dogs had significantly higher PV and PG than CONT group (p<0.001).

**Table-3:** The parameters of echocardiography in control and pulmonic stenosis dogs.

Parameters	CONT group	PS group
IVSdN (cm)	0.47±0.02	0.60±0.03**
IVSsN (cm)	0.60±0.03	0.78±0.05**
LVIDdN (cm)	1.35±0.05	0.95±0.06***
LVIDsN (cm)	0.83±0.05	0.47±0.05***
LVPWdN (cm)	0.40±0.02	0.46±0.03
LVPWsN (cm)	0.60±0.02	0.66±0.03
LAN (cm)	0.80±0.04	0.83±0.05
AON (cm)	0.61±0.03	0.64±0.03
LA:AO	1.34±0.05	1.35±0.10
PV (m/s)	0.90±0.04	5.66±0.40***
PG (mmHg)	3.30±0.32	135.45±16.60***

Data presented as mean±SEM using t-test.

** p<0.01 and

*** p<0.001. CONT=Control, PS=Pulmonic stenosis, IVSdN=Interventricular septum thickness during diastole normalized by body weight, cm=Centimeter, LVIDdN=Left ventricular internal diameter during diastole normalized by body weight, LVPWdN=Left ventricular posterior wall thickness during diastole normalized by body weight, IVSsN=Interventricular septum thickness during systole normalized by body weight, LVIDsN=Left ventricular internal diameter during systole normalized by body weight, LVPWsN=Left ventricular posterior wall thickness during systole normalized by body weight, LAN=Left atrium diameter normalized by body weight, AON=Aorta diameter normalized by body weight, LA:AO=Left atrium to aorta diameter ratio, PV=Pulmonic valve velocity, m/s=Meter per second, PG=Pressure gradient and mmHg=Millimeter mercury

**Figure-1:**
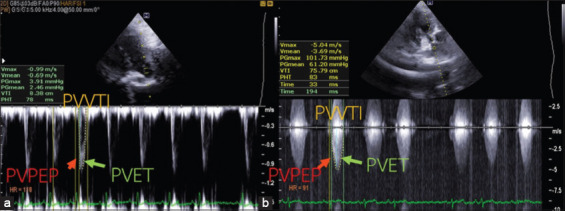
The continuous wave Doppler echocardiographic images showed PVVTI (yellow dot), PVPEP (red arrows) and PVET (green arrows) on the PSAX view at pulmonic valve level using Doppler in (a) Control dog and (b) Dog with pulmonic stenosis. PVVTI=Pulmonic valve velocity time integral, PVPEP=Pulmonic valve pre-ejection period, PVET=Pulmonic valve ejection time, PSAX=Parasternal short axis.

The systolic function parameters are shown in Table-4 while examples of echocardiographic images of STI and VTI are shown in Figure-1. The FS of the left ventricle in PS group was significantly higher than in CONT group (p<0.01). The right-sided systolic function of echocardiographic parameters showed that PS group had significantly increased pulmonic valve VTI (PVVTI) (p<0.001). There was also increased pulmonic valve ET (PVET) (p<0.05) without change in pulmonic valve PEP (PVPEP), causing a reduction of PVPEP to ET ratio (PVPEP:PVET) (p<0.05) in PS compared with CONT group. When considering the left side, no changes in aortic valve VTI (AVVTI) was found. Thus, PVVTI to AVVTI ratio (PVVTI:AVVTI) was significantly higher in PS dogs (p<0.001). Moreover, aortic valve PEP (AVPEP), aortic valve ET (AVET), and aortic valve PEP to ET ratio (AVPEP:AVET) were not significantly different between groups. The RV wall thickness to the left ventricular posterior wall thickness ratio and RA to LA diameter ratio (RA:LA) of PS dogs were significantly higher than those of CONT dogs (p<0.001). The PG values negatively correlated with LVIDdN (r=−0.751, p<0.001, n=25) but positively correlated with PVVTI:AVVTI (r=0.848, p<0.001, n=25).

**Table-4:** Systolic function, severity of the right ventricular hypertrophy and right atrium enlargement parameters in control and pulmonic stenosis dogs.

Parameters	CONT group	PS group
FS (%)	36.12±2.17	49.76±3.88**
PVVTI (cm)	10.07±0.29	80.15±8.32***
AVVTI (cm)	11.19±0.59	10.38±1.02
PVVTI:AVVTI	0.93±0.05	8.67±1.22***
PVPEP (ms)	52.75±2.04	51.00±3.79
PVET (ms)	147.42±8.23	187.08±12.19*
PVPEP:PVET	0.37±0.02	0.29±0.03*
AVPEP (ms)	58.75±4.78	57.31±3.53
AVET (ms)	123.67±7.48	129.85±5.09
AVPEP:AVET	0.48±0.03	0.45±0.04
RVWd:LVWd	0.66±0.03	1.26±0.12***
RA:LA	0.83±0.01	1.32±0.07***

Data presented as mean±SEM using t-test.

* p<0.05,

** p<0.01 and

*** p<0.001. CONT=Control, PS=Pulmonic stenosis, FS=Fractional shortening, PVVTI=Pulmonic valve velocity time integral, cm=Centrimeter, AVVTI=Aortic valve velocity time integral, PVVTI:AVVTI=Pulmonic valve velocity time integral to aortic valve velocity time integral ratio, PVPEP=Pulmonic valve pre-ejection period, ms=Millisecond, PVET=Pulmonic valve ejection time, PVPEP:PVET= Pulmonic valve pre-ejection period to pulmonic valve ejection time ratio, AVPEP=Aortic valve pre-ejection period, AVET=Aortic valve ejection time, AVPEP:AVET= Aortic valve pre-ejection period to aortic valve ejection time ratio, RVFW:LVPW=Right ventricular free wall thickness to left ventricular posterior wall thickness ratio and RA:LA=Right atrium to left atrium diameter ratio

## Discussion

In our study, the PS dogs presented were mostly small to medium breeds. The breeds included Bulldogs, Shih Tzu, Pomeranian, Poodle, and Beagle. Both French and English Bulldogs were over-represented. These results are consistent with the previous studies which showed the high prevalence of PS in Bulldogs [1,8] and may coexist with other cardiac congenital defects [9]. The average age of PS dogs that showed clinical signs has some variations. However, all PS dogs that are brachycephalic breeds were younger than 1 year old. Upper respiratory obstruction commonly found in brachycephalic dogs that can depress pulmonary function may lead to exercise intolerance and other right-sided heart failure signs; in young dogs this may not be detected yet [10]. Males seem to have a higher risk for PS more than females, as was reported earlier [8,11]. However, in other PS dog studies, equal distribution between sexes was reported [3]. In English Bulldogs, the important anatomical variation of the coronary artery is the greatest concern. An aberrant coronary artery that emerges and wraps around the PA will be the limiting factor for PS and treatment options. Reports of English Bulldogs and Boxers with an aberrant coronary artery were published showing variable outcomes [3]. In the present study, one English Bulldog had an aberrant coronary artery and one English Bulldog had both ASD and an aberrant coronary artery, while none of the French Bulldogs had an aberrant coronary artery. It was interesting to see that PS can occur in concomitance with other congenital defects. This study shows four out of 13 dogs had ASD. Two of the dogs with ASD were French Bulldogs and the rest were either English Bulldogs or Pomeranian. In terms of clinical presentation, left-to-right shunting of ASD can lead to right-sided volume overload, and right-to-left shunting of ASD can cause a reduction of oxygenated blood leading to cyanosis [12] and polycythemia. Thus, the clinical signs of PS dogs with ASD may be more severe. In the present study, all four PS dogs with ASD had PG graded as severe along with the clinical signs of exercise intolerance, while polycythemia and syncope were found in only 2 PS dogs with right-to-left shunting of ASD.

Rather than the congenital defects, PS was divided into type A and type B, based on the degree of pulmonic valvular leaflet thickening, PA:AO ratio, and the presence of pulmonary valvular hypoplasia [11]. Our study showed that seven out of 13 PS dogs had type A PS while six PS dogs had type B PS. Other reports showed that the prevalence of type A was higher than type B, with a better outcome after BV [11,13]. The average PG in type A and type B was 103.9±21.2 and 172.2±17.3 mmHg, respectively. Most dogs had valvular PS that was similar to other studies [1,3].

Polycythemia was present in two PS dogs with right-to-left shunting of ASD. The polycythemia should be a result of ASD rather than PS. Polycythemia occurred because of the lack of oxygenated blood due to mixing of low oxygenated blood from RA through the shunt of ASD. Decreased partial pressure of oxygen stimulated erythropoietin production from the kidney and sequentially increased RBC production. Reports of congenital defect causing secondary erythrocytosis of cyanotic congenital heart diseases were found [14].

Most of the clinical signs found in these dogs with PS were exercise intolerance and syncope, as mentioned earlier [11,13]. The syncope was caused by increased right cardiac afterload causing less blood to flow into the PA. In addition, increased RV pressure will limit the venous return to the left side of the heart which will eventually cause low CO and syncope. Four out of 13 PS dogs presented with right-sided heart failure wherein ascites developed afterwards. The medications that were prescribed in PS dogs were included positive inotropes (i.e., pimobendan), afterload reducers such as angiotensin-converting enzyme inhibitors or sildenafil, and preload reducers including furosemide. However, BV is the best choice especially for moderate to severe PS cases [11].

The electrocardiographic patterns in all CONT dogs showed RSA, while almost half of the PS dogs showed ST. The ST may have resulted from the CHF-induced sympathetic compensation, stress, anxiety, and excitement. The shape of the ECG waveform in PS dogs was unique, as previously described in PS dogs [6]. Increased S wave deflection with decreased R:S ratio was more pronounced. The presence of R:S abnormalities were found in ten PS dogs, all of them having moderate and severe PS suggesting that these dogs had hypertrophy, called RV concentric hypertrophy, from increased right-sided pressure overload. Hypertrophy is defined as the cardiac maladaptation with increased cardiac wall thickness and cardiac myocyte mass for increased systolic function as compensation during pressure overload [15]. Increased hypertrophy without muscle elongation and a reduced radius causes tremendous tension of the heart, as described by Laplace’s law. Thus, the increased isometric ventricular pressure was to overcome the increase in afterload at the stenotic area. High shear stress at the right outflow tract may be a reason for developing the gradient between the right and left sides of the ventricle when PS progressed. Another abnormality on ECG is prolonged QRS duration due to increased RV mass. The ECG results of PS dogs in the present study were similar to the cardiac electrical changes in human patients with PS, which showed alterations in axis and QTc compared with those of healthypersons [16]. Moreover, these cardiac electrical changes showed remarkable improvement after BV and related with the hemodynamic, cardiac structural, and functional improvements measured from echocardiographic parameters and cardiac biomarkers, such as pro B-type natriuretic peptide [16].

In our study, two PS dogs with prolongation of QTc (604.5 and 666.3 ms) had sudden cardiac death at 6.5 and 15 months after BV, respectively. The prolongation of QT especially prolongation of the T-wave onset to T-peak component was reported to be related with an increased risk of sudden cardiac death [17]. Higher P wave amplitude, called P pulmonale, was found in PS dogs. The P pulmonale was previously described as having an intense depolarization of the RA. The increased amplitude may be related to the right atrial enlargement as RA:LA >1. Both RA and RV enlargement in PS dogs was also reported to be associated withcardiac death [18].

Echocardiographic data of the left ventricular functions showed that PS dogs had low volume as shown by a reduction in both LVIDdN and LVIDsN. The limited left-sided venous return is due to the right-sided compression and reduction of blood flow through the stenotic valve. The pressure at the right side can transmit through the IVS which was thickened during both systole and diastole. Thickening of the IVS resulted in a further decline in diastolic volume, especially when the PS progressed, as described previously in PS mice models which showed leftward septal shifting [19]. The further the PG progressed, the further the reduction in the left ventricular chamber as seen by a significant negative relationship. Higher FS may not ensure higher cardiac output in this case. Moreover, these cardiac compensations can induce myocardial ischemia, loss of cardiac compliance, and impair systolic and diastolic functions [18].

The systolic function of the right and left sides of the heart was confirmed by echocardiography. Increased VTI but only on the right side suggested that more force was needed to eject blood into the PA. The PEPs of both sides were not significantly different between PS and CONT dogs but the ET was prolonged only on the right side in PS dogs, resulting in a lower PVPEP:PVET in PS dogs. Thus, pressure in the ventricle after depolarization was elevated due to the prolonged ET despite normal valve opening. A longer valve closure time may be due to either increased RV pressure against the valve or increased momentum of blood in the PA rather than the increased contractility. Prolonged ET time also corresponded to the prolonged QRS complex from ECG.

Although the AVVTI, AVPEP, AVET, and AVPEP:AVET of PS dogs were not different than CONT dogs, FS was higher. Neither of these parameters are indicators of stroke volume. Prolonged ET in patients with aortic stenosis was previously reported, which found that ET did not correlate with stroke volume below 100 ml [20]. The stroke volume was limited by a reduced left ventricular chamber volume resulting in low preload, as seen in this study. Unequal changes of VTI between the left and right sides resulted in an increase in PVVTI:AVVTI in PS dogs as compared with CONT. The PG had a positive relationship with PVVTI:AVVTI, which suggests that the severity of PS caused a higher VTI on the right side.

## Conclusion

PS dogs have thickening of RV and IVS with increased PVVTI and PVET. Delayed pulmonic valve closure is crucial to ensure adequate blood flow to the lung. The aortic valve opening and closing times were unchanged although FS was increased. Diminished left ventricular preload due to right-sided compression is the main cause of low cardiac output.

## Authors’ Contributions

CB supervised and designed the study. PT collected all data whereas SDS performed echocardiography. All authors participated in analysis of data. CB and SS wrote and revised the manuscript. All authors read, revised, and approved the final manuscript.
